# Potential Oral Anticancer Therapeutic Agents of Hexahydrocurcumin-Encapsulated Chitosan Nanoparticles against MDA-MB-231 Breast Cancer Cells

**DOI:** 10.3390/pharmaceutics15020472

**Published:** 2023-01-31

**Authors:** Feuangthit N. Sorasitthiyanukarn, Chawanphat Muangnoi, Clinton B. Gomez, Apichart Suksamrarn, Pranee Rojsitthisak, Pornchai Rojsitthisak

**Affiliations:** 1 Metallurgy and Materials Science Research Institute, Chulalongkorn University, Bangkok 10330, Thailand; 2 Center of Excellent in Natural Products for Ageing and Chronic Diseases, Chulalongkorn University, Bangkok 10330, Thailand; 3 Institute of Nutrition, Mahidol University, Nakhon Pathom 73170, Thailand; 4 Department of Industrial Pharmacy, College of Pharmacy, University of the Philippines Manila, Manila 1000, Metro Manila, Philippines; 5 Department of Chemistry and Center of Excellence for Innovation in Chemistry, Faculty of Science, Ramkhamhaeng University, Bangkok 10240, Thailand; 6 Department of Food and Pharmaceutical Chemistry, Faculty of Pharmaceutical Sciences, Chulalongkorn University, Bangkok 10330, Thailand

**Keywords:** hexahydrocurcumin, chitosan nanoparticles, Box–Behnken design, anticancer

## Abstract

Hexahydrocurcumin-encapsulated chitosan nanoparticles (HHC-CS-NPs) were formulated by oil-in-water emulsification and ionotropic gelation and optimized using the Box–Behnken design. The particle size, zeta potential, and encapsulation efficiency of the optimized HHC-CS-NPs were 256 ± 14 nm, 27.3 ± 0.7 mV, and 90.6 ± 1.7%, respectively. The TEM analysis showed a spherical shape and a dense structure with a narrow size distribution. The FT-IR analysis indicated no chemical interaction between the excipients and the drugs in the nanoparticles, but the existence of the drugs was molecularly dispersed in the nanoparticle matrices. The drug release profile showed a preliminary burst release followed by a sustained release under simulated gastrointestinal (GI) and physiological conditions. A stability study suggested that the HHC-CS-NPs were stable under UV light, simulated GI, and body fluids. The in vitro bioaccessibility and bioavailability of the HHC-CS-NPs were 2.2 and 6.1 times higher than those of the HHC solution, respectively. The in vitro evaluation of the antioxidant, anti-inflammatory, and cytotoxic effects of the optimized HHC-CS-NPs demonstrated that the CS-NPs significantly improved the biological activities of HHC in radical scavenging, hemolysis protection activity, anti-protein denaturation, and cytotoxicity against MDA-MB-231 breast cancer cells. Western blot analysis showed that the apoptotic protein expression of Bax, cytochrome C, caspase-3, and caspase-9, were significantly up-regulated, whereas the anti-apoptotic protein Bcl-2 expression was down-regulated in the HHC-CS-NP-treated cells. Our findings suggest that the optimized HHC-CS-NPs can be further developed as an efficient oral treatment for breast cancer.

## 1. Introduction

The World Health Organization (WHO) reported that cancer was the second most significant health issue and the cause of global deaths [1]. Among the different types of cancers, breast cancer is one of the most frightening cancers and accounts for most cancers in Asian countries [2]. Generally, breast cancer (BC) is classified as either estrogen-receptor-positive (ER+) or -negative (ER−). Other types are further classified as luminal A and B, basal-like, and HER2^+^, based on biomarkers such as progesterone receptor (PR) and human epidermal growth factor receptor 2 (HER2) [3,4]. Triple-negative breast cancer (TNBC) such as MDA-MB-231 is responsible for more than 15–20% of cases, and MDA-MB-23 is typically more aggressive and challenging to treat and more likely to recur compared to HER2^+^ breast cancer because of the lack of ER, PR, and HER2. Therefore, TNBC has a low response to therapeutics and is highly invasive [5,6]. Although chemotherapy is regarded as one of the most effective cancer treatments, its adverse effects in such treatments include reduced immunity, decreased leukocyte count, and alopecia areata, which have been received with critical concern [7]. To avoid these adverse effects, curcumin, a well-known polyphenolic compound from the rhizome of *Curcuma longa*, which possesses anticancer activity against various types of cancers, has received considerable attention for development as a novel anticancer agent with fewer adverse effects [8]. Due to the low water solubility and poor bioavailability of curcumin, the use of curcumin metabolites [9] and curcumin-encapsulated nano-formulations [10] has been proposed to overcome the limitations of curcumin. Among the curcumin metabolites, hexahydrocurcumin (HHC) is one of the major metabolites that exhibit similar or more effective chemical stability, bioavailability, and biological activities, such as antioxidant, anticancer, and anti-inflammatory activities, than the parent curcumin in both in vitro and in vivo studies [11]. Nevertheless, HHC has low solubility, resulting in poor absorption and bioavailability. HHC encapsulated in nanostructures, especially polymeric nanoparticles (NPs) based on natural polymers such as chitosan, may be effectively approached to enhance its solubility and bioavailability.

Chitosan (CS) is an *N*-deacetylated derivative of chitin, having the structure of β-[1–4]-linked D-glucosamine (deacetylated unit), which is mainly found in the exoskeleton of marine crustaceans (i.e., shrimps, crabs, and lobsters) [12]. Various properties show that CS is a suitable candidate for fabricating the NPs as a drug delivery system in this study, such as its biocompatible, non-immunogenic, biodegradable, mucoadhesive, and gelation properties [13]. Our previous studies suggested that the CS-NPs containing curcumin prodrug presented better stability and biological activities, including in vitro bioaccessibility and bioavailability, and antioxidant, anti-inflammatory, and anticancer effects than the free curcumin prodrug [14]. However, no investigations have been reported on using CS-NPs as a delivery system for HHC to enhance their physicochemical properties and biological activities. Therefore, it was chosen for further investigation in the current study.

In the present study, we developed and optimized the formulation of HHC-encapsulated CS-NPs (or HHC-CS-NPs) using the Box–Behnken design (BBD). The physicochemical properties, including the drug release profile, stability, in vitro bioaccessibility, and in vitro bioavailability, of the optimized HHC-CS-NPs were characterized. In addition, the biological activities of the HHC-CS-NPs were investigated for their antioxidant and anti-inflammatory effects as well as their cytotoxicity against MDA-MB-231 breast cancer cells.

## 2. Materials and Methods

### 2.1. Materials

Chitosan (CS, 75 kDa, 84.3 ± 0.2% deacetylated) was provided by Marine Bio-Resources Co., Ltd. (Samut Sakhon, Thailand). Sodium triphosphate (TPP), 1,1-diphenyl-2-picrylhydrazyl (DPPH), 3-(4,5-dimethylthiazol-2-yl)-2,5-diphenyltetrazolium bromide (MTT), Pluronic^®^ F-127mucin powder from porcine stomach Type III, α-amylase, porcine lipase Type II and bile extract powder, pepsin from hog stomach, pancreatin from hog pancreas, and albumin from human serum (HSA) and bovine serum (BSA) were purchased from Sigma-Aldrich Co., Ltd. (St. Louis, MO, USA). Fetal bovine serum (FBS) was obtained from Life Science Production (LPS, Bedford, UK). Serum-free Dulbecco’s modified Eagle’s medium (DMEM) was purchased from Invitrogen Cop. (Grand Island, NY, USA). Hexahydrocurcumin (HHC) was synthesized from curcumin (Cur) following the method described by Srimuangwong et al. [15]. Wright-Giemsa dye was purchased from M&P IMPEX Ltd. (Ladkrabang, BKK, Thailand). Analytical grades of acetic acid, acetone, absolute ethanol, hydrogen peroxide, and dimethyl sulfoxide (DMSO) were purchased from Carlo Erba reagents (Val de Reuil, France). Ultrapure water was obtained from the Thermo Scientific Barnstead MicroPure system (Thermo Fisher Scientific Inc., Waltham, MA, USA). Other reagents were used without further purification.

### 2.2. Experimental Design

The Stat-Ease Design-Expert Software (Version 13 Stat-Ease, Inc., Minneapolis, MN, USA), a 15-experiment run of BBD with 3 factors and 3 levels, was chosen to optimize the HHC-CS-NP formulation. The 3 factors include the concentrations of TPP (*X*_1_), Pluronic^®^ F-127 (*X*_2_), and HHC (*X*_3_), which were found to be critical factors in the formulation of HHC-CS-NPs based on the preliminary experiments. As shown in Table 1, these factors were analyzed at the levels of low (−1), medium (0), and high (+1). The particle size (*Y*_1_), zeta potential (*Y*_2_), and encapsulation efficiency (EE) (*Y*_3_) were chosen as the responses in the present study.

### 2.3. Fabrication of HHC-CS-NPs

The HHC-CS-NPs were fabricated by o/w emulsification and ionotropic gelation, as described by Calvo et al. [16], with modifications. Under magnetic stirring for 30 min at 25 °C, 1 mL of ethanolic HHC solutions with various concentrations was added into 21 mL of chitosan solution (2.6 mg/mL in a 1% (*v*/*v*) acetic acid solution) containing different amounts of Pluronic^®^ F-127. A 7 mL TPP solution with different concentrations was subsequently added to the suspension using an NE-1000 automatic syringe pump (New Era Pump Systems, Inc., Farmingdale, NY, USA) at a rate of 20 mL/h and continuously stirred for 90 min at 25 °C to form the HHC-CS-NPs. The resulting NP suspension was equilibrated overnight at 25 °C in the dark to ensure its complete formation and uniform particle size.

### 2.4. Design and Optimization of HHC-CS-NPs

BBD was used to optimize the HHC-CS-NP formulations. The concentrations of TPP, Pluronic^®^ F-127, and HHC were selected as factors at three levels of study, respectively. The particle size, zeta potential, and EE were chosen as the responses, as shown in Table 1. The pattern of the design is presented in Table 2. Various statistical parameters, including the *p*-value, three regression coefficients (*R*^2^, *R*^2^*_adjusted_*, and *R*^2^*_predicted_*), and the lack of fit *F*-value, were employed to adapt the responses to the suitable mathematical model (including the linear, interaction (2FI), and quadratic models, respectively) generated by the BBD. In addition to the best-fit mathematical model, quadratic polynomial response equations with key and interaction factors were generated. Analysis of variance (ANOVA) was used to evaluate the suitability and validity of the model. The 3D response surface (3D-RSM) plots were generated by design, and the optimal formulation was identified using a numerical optimization technique with the desirability function approach. The optimal formulation was chosen based on the highest desirability value (close to 1), and it was quantitatively compared with the results of the experimental values of the responses. Finally, a checkpoint analysis was performed by calculating the percentage error between the model-predicted and the experimental values to confirm the roles of the obtained polynomial response equation and the 3D response surface plots in predicting the responses.

### 2.5. Characterization

#### 2.5.1. Particle Size, Zeta Potential, Morphology, and Drug–Excipients Interaction

The particle size, zeta potential, and polydispersity index (PDI) of the NPs were analyzed by the dynamic light scattering (DLS) technique using the Zetasizer (Nano ZS, Malvern Instruments Ltd., Malvern, Worcestershire, UK). The NP morphology was evaluated by transmission electron microscope (TEM, H-9500, Hitachi High Technology America Inc., Pleasanton, CA, USA). The NP suspension was diluted with ultrapure water at 30× before being dropped onto a copper grid coated with formvar film and allowed to dry at room temperature before analysis. The particle size of the optimized NPs was measured using the ImageJ software version 1.53t (National Institutes of Health (NIH), Bethesda, MD, USA) [17]. The chemical composition and the drug–NP interactions were analyzed using an attenuated total reflectance-Fourier transform infrared spectrometer (ATR-FTIR, Bruker, Billerica, MA, USA). The IR spectra of the samples, including HHC powder, CS-NPs, and HHC-CS-NPs, were measured in the range of 4000 to 400 cm^−1^.

#### 2.5.2. Determination of EE

The HHC content in the HHC-CS-NPs was determined by UV-Vis spectrophotometry, as described in our previous work [14]. The HHC-CS-NP suspensions were centrifugated at 35,000× *g* rpm at 4 °C for 45 min using Hitachi Ultracentrifugation (Model CP100-NX, Hitachi Koki, Tokyo, Japan). The supernatant containing unencapsulated HHC was collected and further analyzed at 280 nm using a UV-Vis spectrophotometer (Cary-60, Agilent Technologies, Santa Clara, CA, USA). The EE of the HHC-CS-NPs was calculated according to Equation (1).

EE (%) = [(*W_i_* − *W_s_*)/*W_i_*] × 100 (1)

where *W_i_* is the initial amount of HHC added to the formulation, and *W_s_* is the amount of HHC found in the supernatant after centrifugation.

### 2.6. In Vitro Drug Release Study

#### 2.6.1. Simulated Gastrointestinal (GI) Conditions

The release profile of HHC from the HHC-CS-NPs under the simulated GI conditions was determined using the dialysis membrane diffusion technique, as previously described by Omer et al. [18], with modifications. Simulated gastrointestinal (SGI) fluids without digestive enzymes, including simulated gastric fluid (SGF) at pH 1.2, intestinal fluid (SIF) at pH 6.8, and colonic acid (SCF) at pH 7.3, were premixed with ethanol (30% *v*/*v*) to serve as the release medium. Ethanol was added to the simulated fluids to increase the solubility of HHC because the solubility of HHC in the simulated fluids is minimal, thus ensuring the sink condition and avoiding the aggregation of HHC [19]. A 20 mL HHC-CS-NP suspension was placed into a dialysis tubing membrane bag (MWCO 14,000 Da, CelluSep^®^ T1, Membrane Filtration Products, Seguin, Texas, USA). The end-sealed dialysis bag was then immersed in 100 mL of dissolution medium. A 20 mL HHC solution at the equivalent HHC concentration in the HHC-CS-NPs was also performed to observe the effect of the NPs on the release profile of HHC. After shaking in SGF for 2 h at 37 °C, 150 rpm, the sample was transferred to SIF and SCF for 3 h each at 150 rpm at 37 °C. Each 2 mL sample was withdrawn at different time intervals (0–8 h), and an equal volume of fresh dissolution medium was suddenly replaced to maintain the sink state. The HHC concentration in the samples was analyzed by UV-Vis spectrophotometry, as described above. The results were expressed as the cumulative release of HHC versus time.

#### 2.6.2. In Simulated Body Fluid Condition

The in vitro release of HHC from the HHC solution or HHC-CS-NPs in a simulated body fluid (SBF, pH 7.4) containing 30% ethanol with or without 1% human serum albumin (HSA) as a dissolution medium [20] was performed by using the dialysis technique with the same protocol as those tested in the simulated GI conditions described above. The HSA was added to the dissolution medium to closely mimic the physiological condition [21]. The amount of HHC released was quantified at pre-determined time intervals (0.5, 1, 2, 4, 8, 12, 24, and 48 h, respectively), and the release data were then fitted to various mathematical models to determine the release mechanism by using DDSolver (an Excel add-in software package). The highest *R*^2^*_adjusted_* and Akaike information criterion (AIC) values with the lowest model selection criterion (MSC) values were used as indicators for selecting the best-fitted kinetic model [22].

### 2.7. Determination of Physicochemical and Biological Stability

#### 2.7.1. Storage Stability

The storage stability of the HHC-CS-NPs was assessed using the ICH guidelines [23] with modifications. The samples were placed separately at 4 °C ± 0.5 and 25 °C ± 0.5 in an amber bottle with a seal. Samples were taken at 5, 15, 30, 45, 60, and 90 days and evaluated for particle size, zeta potential, and EE, as described above.

#### 2.7.2. Light Stability

The stability against UV light of the HHC-CS-NPs and HHC with an HHC concentration equivalent to that of the HHC-CS-NPs was performed following the method described by Li et al. [24], with modifications. The samples were transferred into a clear glass bottle, placed into a light-proof cabinet, and exposed to a 254 nm UV lamp (model TUV 30 W/G30 T8, Philips, Hamburg, Germany) for different time periods (0–150 min) at 25 °C ± 0.5. The gap between the samples and the UV light sources was fixed at 30 cm. At pre-determined times, the samples were taken to evaluate the EE, as described above. The light stability of the samples was assessed by the HHC retention percentage, which can be calculated using Equation (2).

HHC retention (%) = (HHC at each time interval/HHC at initial) × 100(2)

#### 2.7.3. In Vitro Protein Stability

The colloidal stability in the BSA of HHC-CS-NPs was determined using the method described by Suvarna et al. [20], with modifications. The sample was pre-warmed in a water bath at 37 °C for 10 min to mimic the physiological condition and subsequently mixed with the BSA solution (1 mg/mL in PBS, pH 7.4) at the ratio of 1:2 (sample: protein solution, *v*/*v*) and incubated at 37 °C for up to 24 h. The sample was then taken to measure the particle size and EE at predetermined times (0, 1, 6, 12, 18, and 24 h). The morphology of the HHC-CS-NPs after 24 h incubation with BSA was also examined using TEM, as previously described.

### 2.8. In Vitro Bioaccessibility and Bioavailability

The in vitro bioaccessibility and bioavailability of the HHC-CS-NPs or HHC were determined under simulated GI conditions using the protocol previously reported by Shah et al. [25]. In this study, 10 mL of samples were mixed with 10 mL of pre-warmed simulated saliva fluid (phase I: oral phase) with the pH adjusted to 6.8, followed by incubation in a shaking incubator at 37 °C and 100 rpm for 10 min. After the oral phase, 20 mL of derived samples were added to the SGF (phase II: gastric phase, 20 mL), and the pH was adjusted to 2.5 before incubation at the same condition for 2 h. For intestinal phase digestion, the digested samples derived from the gastric phase were adjusted to pH 7.0 before separately adding the bile extract solution (187.5 mg of bile extract in SIF, 4 mL) and CaCl_2_ solution (110 mg in the SIF, 1 mL), respectively (phase III: intestinal phase). The pH of the samples was adjusted to 7.0 before incubation at 37 °C and 100 rpm for 2 h. The digested samples derived from the intestinal phase were centrifuged at 35,000× *g* rpm at 25 °C for 30 min. The HHC in the supernatant was extracted by mixing 10 mL of supernatant with an equivalent volume of chloroform and centrifuged at 35,000× *g* rpm at 25 °C for 10 min. Then, the amount of HHC contained in the chloroform layer was analyzed by UV-Vis spectrophotometry. It was noted that the supernatant containing the HHC liberated from the solid NP matrix was considered to be the micellar fraction, and the micellarized HHC available for absorption was regarded as the bioaccessible fraction. The transformation (T), bioaccessibility (BA), and bioavailability (B) of HHC were calculated using Equations (3)–(5), respectively [26].

T (%) = (*C_Digesta_*/*C_Initial_*) × 100 (3)

BA (%) = (*C_Micelle_*/*C_Digesta_*) × 100 (4)

B (%) = (*C_Micelle_*/*C_Initial_*) × 100 (5)

where T is the transformation that refers to the concentration of HHC that remains in the bioactive forms, and *C_Digesta_* and *C_Initial_* are the concentrations of HHC in the raw digesta and sample, respectively, before being subjected to digestion in the simulated GI model. BA is bioaccessibility, which refers to the HHC fraction available for absorption in the mixed micelles arriving at the small intestine phase. *C_Micelle_* is the concentration of HHC in the micellar phase. B is the bioavailability obtained by the multiplication of the T value and the BA value, which is the ratio between the *C_Micelle_* and the *C_Initial_*.

Notably, bioavailability can only be measured by an in vivo assay of the metabolites existing in blood and/or urine after compound consumption as it has metabolic or physiological endpoints that are almost impossible to factor in an in vitro assay [27]. In addition, several host factors that can potentially influence compound absorption, such as age, genotype, physiological state, infectious disease states, and so on, are impossible to factor in the in vitro method [28]. For this reason, bioavailability can only be measured by in vivo assays as part of human or animal pharmacokinetic studies [29]. However, in vitro bioavailability can serve as an alternative assay for screening and predicting bioavailability because the method is less expensive, faster, and offers better control of the experimental factors [30]. The in vitro bioavailability results are also useful for further evaluating the in vivo bioavailability in pre-clinical and clinical studies.

### 2.9. In Vitro Antioxidant Capacity

#### 2.9.1. DDPH Scavenging Activity

The DPPH scavenging activity of HHC-CS-NPs or unencapsulated HHC was determined following the method previously described by Navarro-Hoyos et al. [31], with modifications. One milliliter of the sample with different HHC concentrations (5–80 μg/mL) was mixed with 0.5 mL of an ethanolic DPPH solution (0.25 mM). The sample was then incubated in a dark place at 25 °C. After 30 min incubation, the absorbance of the sample was measured by a microplate reader spectrophotometer (BMG LABTECH, Ortenberg, Germany) at 517 nm. Ethanol (1 mL) and ethanolic DPPH solutions (0.5 mL) without HHC were used as the control samples, and ethanol served as a blank. The DPPH scavenging activity from the sample was calculated using Equation (6).

DPPH scavenging activity (%) = [(*A_control_* − *A_sample_*)/*A_control_*] × 100 (6)

where *A_control_* and *A_sample_* are the absorbance of the control and the tested samples, respectively.

The inhibition curve between the DPPH scavenging activity and the sample concentration was plotted to estimate the efficient concentration at which 50% oxidation occurs (EC_50_). The EC_50_ is the sample concentration required to reduce the initial DPPH concentration by 50% and is expressed in μg/mL [32].

#### 2.9.2. Hydroxyl (OH•) Scavenging Assay

The OH• radical scavenging activity of the HHC-CS-NPs or the unencapsulated HHC was performed according to the protocol reported by Lei et al. [33], with modifications. The sample group (*A_s_*) solution (1 mL HHC-CS-NPs or HHC solution with an HHC concentration of 5–80 μg/mL, 2 mL of ethanolic 1,10-phenanthroline solution, 2 mL 0.01 M PBS (pH 7.4), and 2 mL of 0.75 mM of ferrous sulfate solution (0.114 g FeSO_4_·2H_2_O dissolved in 1 L of ultrapure water)) was mixed with 1 mL of 0.01% (*v*/*v*) H_2_O_2_ solution and incubated at 37 °C for 1 h. A microplate reader spectrophotometer (BMG LABTECH, Ortenberg, Germany) was used to measure the sample’s absorbance at 536 nm. The damage (*A_d_*) and non-damage (*A_n_*) groups were carried out under the same conditions as the sample group, except that for the damage group 1 mL of ultrapure water was used instead of the sample, and for the non-damage group, 1 mL of ultrapure water was used instead of H_2_O_2_. The OH• radical scavenging activity from the sample was calculated using the following Equation (7).

Hydroxyl radical scavenging activity (%) = [(*A_s_* − *A_d_*)/(*A_n_* − *A_d_*)] × 100 (7)

where *A_s_*, *A_d_*, and *A_n_* referred to the absorbance of the sample, damaged group, and non-damaged group, respectively.

### 2.10. In Vitro Anti-Inflammatory Activity

#### 2.10.1. Red Blood Cells Membrane Stabilization

The in vitro anti-inflammatory activity of the HHC-CS-NPs and unencapsulated HHC was determined using a hemolysis assay, as previously described by Jiang et al. [34], with modifications. Briefly, sodium citrate-stabilized male Wistar rat (2 weeks’ age, 350–400 g weight) blood samples were collected and provided by the National Laboratory Animal Center of Mahidol University, Thailand. The animal research protocol was approved by the National Laboratory Animal Center Animal Care and Use Committee (NLAC-ACUC), National Laboratory Animal Center, Mahidol University, Thailand (approval code: RA2022-09; approval date: 28 January 2022). Whole blood was centrifuged at 3500× *g* rpm for 5 min to separate the packed erythrocytes (RBCs) from the plasma. To prepare the RBC suspension, 100 μL of packed RBCs were diluted with 0.9 mL PBS (pH 7.4) to prepare the RBC suspensions. The RBC suspensions were then individually mixed with 0.8 mL samples of the HHC-CS-NPs or HHC at an equivalent HHC concentration of 20–100 μg/mL. Then, 0.8 mL ultrapure water or PBS (pH 7.4) was added to 0.2 mL diluted RBC suspensions as the positive and negative control groups, respectively. All the samples were vortexed and incubated at 37 °C for 30 min before being centrifuged at 3500× *g* rpm for 5 min. Furthermore, 100 μL supernatant was collected and transferred to a 96-well plate, and the absorbance of hemoglobin was measured by a microplate reader at 577 nm. The hemolysis percentage was calculated according to Equation (8).

Hemolysis (%) = [(*A_s_* − *A_n_*)/(*A_p_* − *A_n_*)] × 100% (8)

where *A_s_*, *A_n_*, and *A_p_* represented the absorbance of the sample, the negative control, and the positive control group, respectively. The in vitro anti-inflammatory activity is the hemolysis protection percentage, calculated by the percentage of hemolysis protection = 100—hemolysis percentage [34].

To affirm the hemolysis protection activity of the NPs, the morphology of the RBCs was determined according to the method previously described by Jiang et al. [34], with modifications. A 0.2 mL diluted RBC suspension was mixed with a 0.8 mL sample and incubated for 30 min at 37 °C. Then, the mixture was dropped onto a glass slide to make a blood smear and fixed with methyl alcohol. The slide was stained with Wright-Giemsa dye and examined under the inverted optical microscope (Olympus LX51, Tokyo, Japan). The magnification was 40×.

#### 2.10.2. Protein Denaturation Determination

The effect of the HHC-CS-NPs or the unencapsulated HHC on the protein denaturation was determined by the protein denaturation assay according to the protocol described by Gunathilake et al. [35], with modifications. In brief, 5 mL of each sample consisting of 0.2 mL of BSA (1% *w*/*v*), 4.78 mL of PBS (pH 6.4), and 0.02 mL of HHC-CS-NPs or HHC solution at an equivalent HHC concentration of 20–100 μg/mL were incubated in a water bath at 37 °C for 15 min. After incubation, denaturation was induced by heating; the samples were heated at 70 °C for 5 min and cooled at room temperature. Then, the sample was measured using a microplate reader spectrophotometer at 660 nm. PBS (pH 6.4) was used as the control throughout the experiment. The inhibition of protein denaturation percentage was calculated by using Equation (9).

Inhibition of protein denaturation (%) = 100 × (1 − *A_s_*/*A_c_*) (9)

where *A_c_* is the absorbance of the control sample, and *A_s_* is the absorbance of the tested sample.

### 2.11. In Vitro Cytotoxicity

The cytotoxicity of the blank NPs without HHC (CS-NPs), the unencapsulated HHC, and the HHC-CS-NPs against MDA-MB-231 breast cancer cells (ATCC, Manassas, VA, USA) was determined by the MTT assay, as previously reported by Muangnoi et al. [36], with modifications. Briefly, the breast cancer cells were cultured in a complete medium (CM) comprising DMEM supplemented with 10% (*v*/*v*) heat-inactivated fetal bovine serum (FBS) and 1% (*v*/*v*) penicillin/streptomycin. The cultured cells were maintained in a humidified atmosphere of 95:5 (*v*/*v*) air:CO_2_ at 37 °C. The cells were seeded at a cell density of 1 × 10^4^ cells/well/200 μL in a standard 96-well plate, grown for 24 h to obtain a confluent monolayer, and used for further experiments.

#### 2.11.1. Evaluation of Cytotoxicity of CS-NPs

The cytotoxicity of the blank NPs without HHC (CS-NPs), the unencapsulated HHC, and the HHC-CS-NPs against the MDA-MB-231 breast cancer cells (ATCC, Manassas, VA, USA) was determined by the MTT assay, as previously reported by Muangnoi et al. [36], with modifications. Briefly, the breast cancer cells were cultured in a complete medium (CM) comprising DMEM supplemented with 10% (*v*/*v*) heat-inactivated fetal bovine serum (FBS) and 1% (*v*/*v*) penicillin/streptomycin. The cultured cells were maintained in a humidified atmosphere of 95:5 (*v*/*v*) air:CO_2_ at 37 °C. The cells were seeded at a cell density of 1 × 10^4^ cells/well/200 μL in a standard 96-well plate, grown for 24 h to obtain a confluent monolayer, and used for further experiments.

#### 2.11.2. Evaluation of Cytotoxicity of CS-NPs

After incubation for 24 h, the culture medium was removed, and the cells were washed with serum-free DMEM. The 200 µL of DMEM containing CS-NPs at different content percentages (5, 10, 20, 50, and 100%, respectively) was then immediately added into the cells and incubated at 37 °C for 24 h. Afterward, the sample was removed, and the cells were washed with PBS and incubated with an MTT solution (0.5 mg/mL in PBS) for 4 h. The DMEM was used as a control. After incubation, the MTT solution was removed, and DMSO was added to the cells to dissolve the formazan crystals. The optical density of the solution was measured using a microplate reader spectrophotometer at 540 nm. The cell viability was calculated with Equation (10).

Cell viability (%) = (*OD_sample_*/*OD_control_*) × 100 (10)

The non-toxicity dose of the CS-NPs compared with the control (*p* > 0.05) was selected for the cell viability assay of the HHC-CS-NPs or the unencapsulated HHC in further experiments.

#### 2.11.3. Evaluation of Cytotoxicity of HHCNPs

The cytotoxicity effect against the MDA-MB-231 breast cancer cells of the HHC-CS-NPs or the unencapsulated HHC at the equivalent HHC concentrations in the range of 5–10 µg/mL was performed by the MTT assay following the same protocol as that used with the CS-NPs, as described above.

### 2.12. Western Blot Analysis

The expression level of the apoptotic and anti-apoptosis proteins in a sample of the HHC-CS-NPs or unencapsulated HHC-treated cells was performed by a previous protocol described by Muangnoi et al. [36], with modifications. Briefly, the MDA-MB-231 breast cancer cells were seeded at a density of 1.0 × 10^6^ cells/well in 6-well cell culture plates and incubated for 24 h. After incubation, the cells were washed twice with the serum-free medium (free phenol red) and treated with the sample for 24 h. Then, the cells were re-suspended in an ice-cold hypotonic lysis buffer for 30 min at 4 °C to extract whole cellular proteins. The lysates were centrifuged at 13,500× *g* rpm at 4 °C for 5 min. An equivalent amount of each protein sample (40 µg) was added to 10% sodium dodecyl sulfate polyacrylamide gels (SDS-PAGE) and placed for electrophoresis. After transferring the target protein samples from the gel to a pure nitrocellulose membrane (Amersham™Protran^®^, Sigma Aldrich), they were blocked with 5% dry milk at 25 °C. The membranes were treated overnight at 4 °C with the specific primary antibodies: Bcl-2 (1:1000), Bax (1:1000), cytochrome C (1:1000), or β-actin (1:5000). The membranes were washed in triplicate with tris-buffered saline (TBS) and polysorbate-20 before being treated for 2 h with a species-specific horseradish peroxidase (HRP) conjugated secondary antibody reacted with Super SignalTM solution (Endogen Inc., Rockford, IL, USA). The membrane was then subjected to an X-ray film; the attached antibody was removed, and the protein loading was confirmed using an anti-*β* actin antibody. ImageJ software was used to quantify the density of the protein target bands. The results were expressed as a ratio of the band intensities of the target proteins and the *β*-actin. Quantification of the protein target band density was performed using ImageJ software. The results were expressed as a relative ratio of the band intensities of the target proteins and the *β*-actin.

### 2.13. Caspase-3 and -9 Activities Analysis

The cells were homogenized in a hypotonic buffer to obtain the part of the supernatant. The supernatant was added to a specific substrate (N-acetyl-Asp-Glu-Val-Asp-p-nitroanilide or N-acetyl-Leu-Glu-His-Asp-p-nitroanilide for caspase-3 or caspase-9, respectively) at the concentration of 100 µmol/L. The mixture was then incubated at 37 °C for 1 h prior to absorbance measurement at 450 nm using the microplate reader.

### 2.14. Statistical Analysis

All the experiments were performed in triplicate, and the data are expressed as the mean ± standard deviation (SD). Statistical analysis was performed with one-way ANOVA (between groups) and a *t*-test (within groups) using Microsoft Excel 365 version 2212, accepting significance at the *p* < 0.05 level.

## 3. Results and Discussion

### 3.1. Statistical Analysis of the BBD

The responses of particle size (*Y*_1_), zeta potential (*Y*_2_), and EE (*Y*_3_) were separately fitted to linear, interaction (2FI), and quadratic mathematical models using linear regression to obtain the model of choice with the statistically significant (*p* ˂ 0.05) and *R*^2^ and *R*^2^*_adjusted_*, and *R*^2^*_predicted_* values close to one. ANOVA analysis was carried out to identify the statistically significant model terms of the selected model on the responses. The model terms with *p*-values < 0.05 are statistically significant. As shown in Table S1 (see Supplementary), the linear model with the above criteria was chosen for particle size and zeta potential, while EE followed the quadratic model. The final model regression equations for the responses related to the different factors and interactions expressed in terms of coded variables were achieved using Design Expert^®^ software and are shown in Equations (S1)–(S3) (see Supplementary). The value and sign of the regression coefficient (positive or negative) in front of each factor in the regression equation are able to indicate the majority factor and its effect on the response.

#### 3.1.1. Effect of Factors on Particle Size

**Figure 1:**
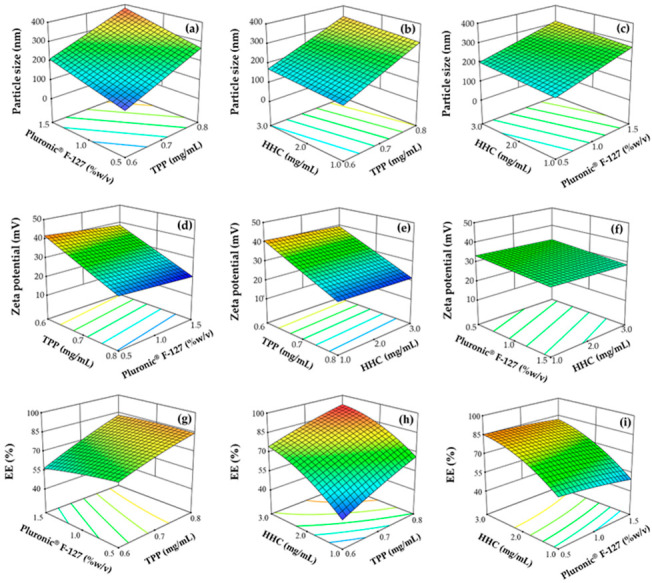
The 3D response surface plot shows the effect of the factors, including TPP, Pluronic^®^ F-127, and HHC concentrations, on particle size (**a**–**c**), zeta potential (**d**–**f**), and EE (**g**–**i**).

The NPs with a particle size range of approximately 40 to 400 nm are appropriate to ensure a long circulation time and an enhanced drug accumulation in the cancer cells with reduced renal clearance [37]. As shown in Table 2, the particle sizes of the HHC-CS-NPs ranged from 102 ± 15 nm to 387 ± 21 nm, suggesting the capacity to produce NPs within an appropriate size range. The ANOVA analysis indicated that all three factors were statistically significant factors that influenced the particle size (*p* < 0.05). The model regression equation, as shown in Equation (S1), indicates the positive influence of all the factors on the response, and the TPP concentration (*X*_1_) was the major factor that had the strongest effect on the increase in particle size compared to the other factors. The 3D-RSM plot in Figure 1a–c shows that increasing the concentrations of TPP, Pluronic^®^ F-127, or HHC increased the particle size. The highest particle size (387 ± 21 nm) was obtained when the TPP and Pluronic^®^ F-127 were used at a high level (Table 2). At a high level of TPP, the CS concentration was insufficient to form an effective electrostatic cross-linkage between the positively charged amino group of the CS and an anionic group of the TPP. This led to the accumulation of excess TPP on the surface of the NP and an increase in particle size [38]. A significant increase in particle size was also observed as an increase in the Pluronic^®^ F-127 (*X*_2_) or HHC (*X*_3_) concentration in the formulation. This may be due to an increase in the number of cooperating molecules in the NPs since an increase in the initial amount of formulation compositions results in an increase in the particle size [39].

#### 3.1.2. Effect of Factors on Zeta Potential

Theoretically, the potential colloidal stability of NPs is strongly associated with the zeta potential [40]. A high zeta potential value indicates the high stability of the system. The values between ±20 and ±40 mV provide the system’s stability and have less tendency to form aggregates or an increase in particle size due to all the particles in the system repelling each other by the effect of high negative or positive zeta potential in the system [41]. As shown in Table 2, the zeta potential of the formulated HHC-CS-NPs ranged from 20.7 ± 0.4 mV to 43.8 ± 0.2 mV, suggesting the good physicochemical stability of the prepared NPs. The ANOVA analysis revealed that the zeta potential was significantly affected by TPP (*X*_1_) and Pluronic^®^ F-127 (*X*_2_) (*p* < 0.05), while the HHC concentration (*X*_3_) had a non-significant effect (*p* ˃ 0.05). The model regression equation, as shown in Equation (S2), indicates that all the factors had a negative effect on the zeta potential, with the TPP concentration being the major factor that had the strongest effect on the zeta potential compared to other factors. The effects of TPP and Pluronic^®^ F-127 were demonstrated by the 3D-RSM plot shown in Figure 1d–f. The zeta potential of the NPs was significantly decreased, which accordingly increased the TPP concentration due to the neutralization of the NH^+^_3_ group of CS by the negative ions of TPP [42]. It was also observed that an increased Pluronic^®^ F-127 concentration led to a significant decrease in the zeta potential due to the neutralization of Pluronic^®^ F-127 by the polymer network and the higher system viscosity, which causes the colloidal instability leading to unstable particle aggregation [43].

#### 3.1.3. Effect of Factors on EE

The EE of the HHC-CS-NPs ranged from 43.4 ± 1.8% to 91.2 ± 0.8%, as shown in Table 2, suggesting that HHC was highly encapsulated by the CS-NPs. The ANOVA analysis indicated that all the factors significantly affected the EE of the prepared NPs (*p* < 0.05). Based on the model regression equation, as shown in Equation (S3), the TPP (*X*_1_) and HHC (*X*_3_) concentrations had a positive effect on the EE. In contrast, the Pluronic^®^ F-127 concentration (*X*_2_) had a negative effect, with the HHC concentration being the major factor affecting the EE. The effect of all the factors is represented by the 3D-RSM plot in Figure 1g–i. The EE was significantly increased by increasing the HHC and TPP concentrations, whereas the increases in the Pluronic^®^ F-127 concentration were related to a significant decrease in the EE. An increasing initial amount of HHC in the formulation led to an increase in the drug molecules encapsulated in the 3D lattice structure of the ionic gelated CS-NPs, resulting in an increase in the EE [44]. A higher TPP concentration could gelate a larger amount of CS, and a corresponding amount of drug could be encapsulated in the NPs [42]. In contrast, the EE was decreased by increasing the Pluronic^®^ F-127 concentration. This might be due to the higher system viscosity, which caused particle aggregation and decreased EE.

### 3.2. Optimization and Model Validation

The factors were optimized using the numerical optimization method with the desirability function provided by the Design Expert^®^ software. The optimized formulation was selected based on the criteria shown in Table 1. The composition of the optimized formulation was 0.75 mg/mL TPP (*X*_1_), 0.5% (*w*/*v*) Pluronic^®^ F-127 (*X*_2_), and 3 mg/mL HHC (*X*_3_). The predicted values of the responses were a particle size (*Y*_1_) of 250 nm, a zeta potential (*Y*_2_) of 26.8 mV, and an EE (*Y*_3_) of 88.7%. After the fabrication of the HHC-CS-NPs using the optimized condition, the observed values of the characteristics were the particle size of 256 ± 14 nm, the zeta potential of 27.3 ± 0.7 mV, and the EE of 90.6 ± 1.7%. The composition check-point formulation with a percentage error between the predicted and the observed values was relatively low, with a desirability value close to 1 (0.95%), as demonstrated in Table 3. Furthermore, no statistical significance (*p* > 0.05) between the predicted and observed values was observed, indicating the good reliability of the model. Therefore, this statistically optimized condition could be used for the preparation of HHC-CS-NPs.

### 3.3. Characterizations

**Figure 2:**
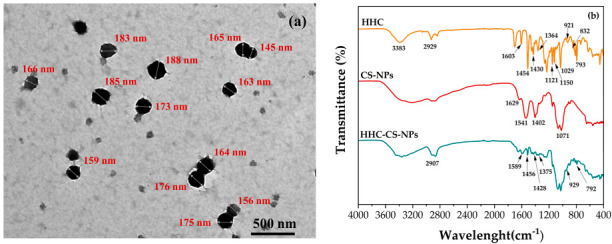
(**a**) TEM analysis of optimized HHC-CS-NPs (at 15,000× magnification) and (**b**) FTIR spectra of HHC, CS-NPs, and optimized HHC-CS-NPs.

The particle size, zeta potential, and EE of the optimized HHC-CS-NP formulation are shown in Table 3, and the polydispersity index (PDI) is 0.43 ± 0.4. The PDI value is related to the size distribution of the NPs, which ranges from zero to 1. The values close to zero show homogeneous NPs, while a value above 0.5 indicates heterogeneous NPs [45,46]. The optimized HHC-CS-NPs show PDI values of less than 0.5, which are considered good particle size distributions. The results are in agreement with the study reported by Lino et al. [47]. The morphology of the optimized HHC-CS-NPs visualized by TEM shows that they are spherical in shape and have a solid and dense structure (Figure 2a). The particle size obtained from the ImageJ software was 181 ± 12 nm, indicating good particle size distribution. These results correlated well with the polydispersity index (PDI) value results obtained from the DLS analysis. In general, the lower PDI value (close to zero) has long been used as a criterion for indicating a formulation with perfectly uniform mono-dispersion. Rosyada et al. [45] and Avadi et al. [46] also suggested that a PDI value above 0.5 indicates heterogeneous NPs. Therefore, the optimized HHC-CS-NPs with the PDI value of 0.43 ± 0.4 could be considered good particle size distributions. The results were in agreement with the study reported by Lino et al. [47]. However, the particle size determined by TEM was smaller than that determined by DLS (256 ± 14 nm). This is because the TEM image was obtained from a dry sample under vacuum and provided the actual radius of the particles, whereas the DLS profile was taken in an aqueous solution [48,49]. Bao et al. [50] demonstrated that particles in the range of 50 to 200 nm with a spherical shape and a solid dense structure have the highest tendency for long circulation and exhibit efficient extravasation into leaky tumor vasculature and accumulation in tumor tissues through enhanced permeability and retention (EPR) effects. Thus, it might suggest that the developed NPs could be a good choice as an effective delivery system for cancer treatment. The FTIR spectra of the HHC, CS-NPs, and optimized HHC-CS-NPs are shown in Figure 2b. The characteristic peaks for pure HHC at 3338, 2929, and 1603 cm^−1^ correspond to O-H stretching, CH_2_ asymmetric stretching, and C=O stretching, respectively. The peaks at 1454, 1430, and 1364 cm^−1^ indicate C=C stretching, C-H bending, and CH_3_ bending, respectively. The peaks at 1150 and 1029 cm^−1^ are attributed to C-O-C stretching. The peak at 1121 cm^−1^ is referred to the C-O stretching [51]. The peaks at 792, 823, and 921 cm^−1^ indicate the bending vibrations of the -CH bond of the alkene group [52]. The FTIR spectrum of the CS-NPs has six characteristic peaks at 3197, 1629, 1625, and 1528 cm^−1^, which are attributed to the O-NH_2_ and –OH group stretching, an interaction between the NH^+^_3_ groups of CS and the phosphate groups of TPP, the CONH_2_ group, the NH_2_ group, and the P=O stretching from the phosphate groups, respectively [53]. The FT-IR spectra of the HHC-CS-NPs showed most of the characteristic peaks of HHC, with some broadening and reduced intensity, indicating that there was no chemical interaction between the HHC and CS-NPs and the presence of the drug as a molecular dispersion in the nanoparticle matrices via physical interaction. The prepared HHC-CS-NPs can maintain the integrity of the HHC structure without causing degradation [54].

### 3.4. In Vitro Release Studies

**Figure 3:**
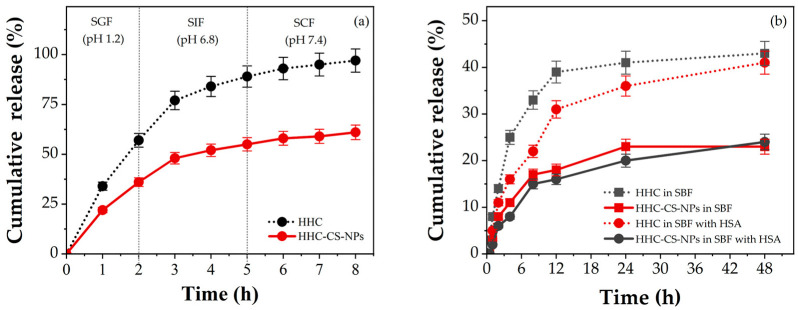
Cumulative release of HHC from HHC solution and HHC-CS-NPs in (**a**) simulated GI fluid and (**b**) simulated body fluid with or without 1% HSA.

It is known that the total GI transit time for drugs or active compounds may vary from one patient to another depending on their physiological conditions [55]. In most healthy cases, the drug carrier is directly transferred to the stomach site after the oral site and stays for a period of 2 to 4 h. They move into the small and large intestine (colon) sites, where they stay for about 3 h each [18]. Figure 3a shows the in vitro HHC release profiles from the optimized HHC-CS-NP formulation under the simulated GI conditions as compared to free HHC. An initial burst release of HHC from the HHC solution or HHC-CS-NPs during the first 1 h of the SGF occurred, with a release of 34% and 22%, respectively. The initial burst release of HHC from the HHC-CS-NPs may be due to the fraction of the HHC, which is adsorbed or weakly bound to the surface of the NPs [56]. At the end of incubation in the SGF, the HHC released from the HHC-CS-NPs (36%) was 1.7 times lower than that from the solution (60%). However, the fast HHC release from the HHC-CS-NPs in the SGF was observed. This could be due to the high swelling of the NP matrix due to the high protonation of the amine group of CS in the acidic pH of the SGF [57]. At the time of incubation in the SIF, the release of HHC from the solution or HHC-CS-NPs was continuously released, but the release rate from the HHC-CS-NPs was observed to be decreased, especially after 3 h. Then, 32% of the HHC from the solution was released throughout the incubation period in the SIF, whereas only 19% of the encapsulated one was released, accounting for only 17% of the former release. The HHC release in SCF was shown to have a similar trend to SIF. The HHC release from the solution and the HHC-CS-NPs was 8% and 6%, respectively. Likewise, our previous study and other studies reported that the CS-NPs showed a lower release of hydrophobic compounds, including astaxanthin, curcumin prodrug, and genistein in high-pH dissolution media (SIF, SCF) than in lower-pH media (SGF), due to the solubility of CS in the different media, which indicates the prolonged degradation of the encapsulants in the high-pH media of the NPs, leading to a sustained release of the encapsulants [14,58]. The results may suggest that the developed NPs showed the controlled and sustained release of HHC in simulated GI conditions.

The in vitro drug release of HHC from the HHC-CS-NPs in simulated body fluid (SBF, pH 7.4) with or without 1% HSA, which mimics the simulated physiological condition, was performed to assess the potential use of the developed NP formulation in preclinical testing prior to in vivo testing, and the results are shown in Figure 3b. Herein, the initial burst release of HHC from the HHC-CS-NPs and the solution in both dissolution media was observed within the first 6 h, and after that, the release was sustained. At 48 h, the HHC released from the HHC-CS-NPs in the SBF and SBF-HSA was 1.7 and 1.9 times lower than the HHC released from the solution, respectively, indicating that the CS-NPs can retard the HHC release more effectively compared to the free HHC. Furthermore, it was observed that the HHC released from the solution in SBF was significantly higher than that in the SBF-HSA (*p* ˂ 0.05), i.e., at 8 h, the HHC released in the SBF and SBF-HSA was 33% and 21%, respectively. Studies have reported that the presence of protein serum in a dissolution medium may increase the system viscosity and that the path lengths for drug diffusion cause a reduction in drug release [59]. The binding between the drug molecules and the serum proteins, forming a large complex that could not pass across the dialysis membrane, may be another reason for the reduced drug release [60]. A similar trend was also observed in the HHC released from the HHC-CS-NPs in the SBF or SBF-HSA. The non-significant difference (*p* ˃ 0.05) indicated the protective effect of CS-NPs against the binding and degradation of the serum proteins, leading to the sustained release of HHC.

It is noted that the presence of ethanol in the release medium used in this release test may not accurately reflect the actual condition of human GI and the physiological environments. For hydrophobic drugs, choosing a release medium to ensure the sink condition has been challenging. Surfactants such as sodium lauryl sulfate (SLS), cetyltrimethylammonium bromide (CTAB), or polysorbate 80 and organic solvents such as ethanol, isopropyl alcohol, or acetone) are typically added to release media to enhance the drug solubility of the sink condition in in vitro drug release tests [61]. Several recent studies have reported the use of different release media containing ethanol up to 50% (*v*/*v*) to investigate the release profile of hydrophobic drugs [62,63,64,65,66,67,68,69,70,71,72,73]. Nasra et al. [64] reported that the release medium containing 50% (*v*/*v*) ethanol is the most suitable for curcumin release (100% release) compared to the other release media containing various types of surfactants such as 0.5–1% *w*/*v* SLS or 30% *w*/*v* polyethylene glycol 400 (PEG 400). Nasra et al. [64] suggested the use of release media containing a high concentration of ethanol to ensure the release of hydrophobic compounds such as curcumin through the dialysis membrane. Therefore, the release medium containing ethanol can be used to investigate the drug release profile of hydrophobic drugs under conditions that mimic the GI and physiological environments. In the case of HHC, one of the major curcumin metabolites, its partition coefficient (Log P) was about 2.7, close to that of curcumin (Log P = 3.2), indicating its low water solubility. Therefore, a release medium containing ethanol was used in the in vitro release study of HHC from the HHC-CS-NPs. The data on the release profile of HHC from the HHC-CS-NPs in release media that reflect the GI and body fluid pH are advantageous and can be helpful in the development of other HHC formulations in future investigations.

According to the kinetic model fitting results, as shown in Table 4, the highest *R*^2^*_adjusted_* and MSC values, as well as the lowest AIC values, were observed for the Peppas–Sahlin model for the in vitro release profile of HHC from the HHC-CS-NPs in both SBF and SBF + 1% HSA. This model describes the combination of Fickian (diffusion-controlled release) and non-Fickian release (the relaxation of the nanoparticle matrix). A value of the release exponent of the Peppas–Sahlin model (m) < 0.45 indicates Fickian diffusion, while a value of 0.45 < m < 0.85 indicates that the drug release is taking place through non-Fickian diffusion [74,75]. In this study, the release exponent value (m) of HHC released in SBF (0.679) or SBF + 1% HSA (0.699) was greater than 0.45 but less than 0.85, indicating that the release of HHC shows a non-Fickian diffusion (anomalous diffusion) mechanism. In addition, the diffusion (*k*_1_) and relaxation (*k*_2_) constants of the Peppas–Sahlin model were compared to explore the contribution of the relaxation of the nanoparticle matrix and the drug diffusion phenomena. A value of *k*_1_ > *k*_2_ suggests that drug diffusion is more important than the relaxation of the nanoparticle matrix. Conversely, a value of *k*_2_ > *k*_1_ implies that the relaxation of the nanoparticle matrix is the dominant contributor [76]. The *k*_1_ and *k*_2_ values in Table 4 suggest that drug diffusion is the predominant contributor to HHC release from the HHC-CS-NPs in SBF and SBF+ 1%HSA. Therefore, the HHC released from the HHC-CS-NPs in both media may occur via two phenomena: (1) the concentration gradient of the drug between the nanoparticles and the release medium (Fickian diffusion) and (2) the erosion of the nanoparticle matrix (non-Fickian diffusion or anomalous transport).

### 3.5. Stability Studies

**Figure 4:**
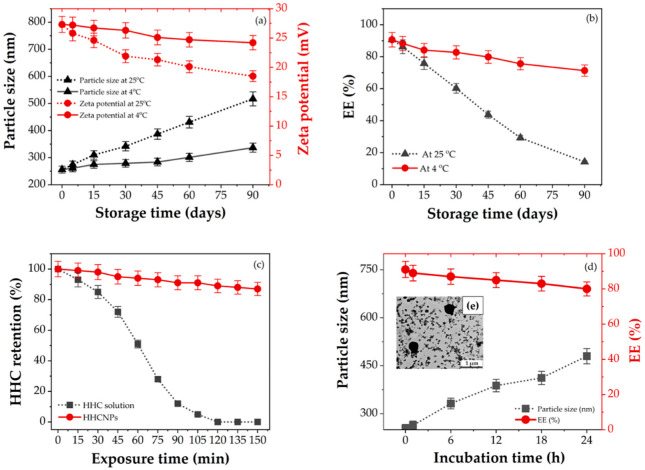
The storage stability of HHC-CS-NPs is expressed in changes of (**a**) particle size and zeta potential and (**b**) EE at 4 °C and 25 °C for 90 days (*n* = 3); (**c**) percentages of retention HHC in HHC-CS-NPs and HHC solution upon exposure to UV light radiation (*n* = 3); (**d**) protein stability expressed in the change in particle size and EE of HHC-CS-NPs after incubation with BSA at 37 °C; (**e**) TEM analysis of HHC-CS-NPs after 24 h incubation with BSA (at 10,000× magnification).

Colloidal delivery systems such as polysaccharide-based carriers must remain stable during the storage and shelf-life of drugs or active compound formulations [77]. Thus, it is essential to investigate the physicochemical stability of the optimized HHC-CS-NPs. The changes in particle size, zeta potential, and EE of the NPs were characterized during storage at 4 and 25 °C for 90 days, and the results are shown in Figure 4a–c, respectively. The particles were unstable at 25 °C and grew after 15 days (˃312 nm), which was significantly different from the first day (~260 nm) (*p* < 0.05). The particle size increased to high values (˃520 nm) during the 90-day period, and particle aggregation was observed, indicating an unstable colloidal suspension (Figure 4a). However, the particle size of the HHC-CS-NPs during the 60 days at 4 °C did not change significantly when compared to the first day (*p* > 0.05), but it increased significantly (*p* < 0.05) after 90 days (Figure 4a), reaching about 330 nm, which is about 1.2 times the initial particle size. The results may indicate that the particle growth was slower when the HHC-CS-NPs were stored at 4 °C. At higher temperatures (25 °C), the kinetic energy of the colloidal system increased, leading to increased particle collisions, resulting in unstable aggregate formulation and an increase in the particle size [78]. As shown in Figure 4a, the increase in particle size in both storage conditions was correlated with a decrease in the zeta potential of the HHC-CS-NPs. The NPs were less stable in the colloidal system when stored at 25 °C, as evidenced by a faster decrease in zeta potential compared to the HHC-CS-NPs stored at 4 °C. During the 90 days of storage, the zeta potential of the HHC-CS-NPs stored at 4 °C and 25 °C was about +24 mV and +18 mV, respectively. When the colloidal system’s zeta potential is low (≤±20 mV), the repulsive force falls behind the attractive forces, resulting in a relatively low-stability system [41]. As shown in Figure 4b, the EE of the HHC-CS-NPs was significantly reduced from the initial level (~91%) after storage at 25 °C for 30 days (<60%) (*p* < 0.05), whereas the EE of the HHC-CS-NPs stored at 4 °C for > 60 days showed no significant change from the initial level (*p* > 0.05). This might be due to the degradation of HHC being fast at high temperatures, resulting in decreases in EE. In addition, the optimized HHC-CS-NPs had better storage stability at 4 °C for up to 90 days. Figure 4c represents the stability of the HHC-CS-NPs and free HHC against UV light exposure. During 60 min of UV light exposure, the free HHC was significantly degraded (50% degraded), whereas the HHC in the HHC-CS-NPs was not significantly degraded (4% degraded) compared to the initial level (*p* > 0.05). In addition, after 150 min of exposure, about 95% of the free HHC and 10% of the HHC-CS-NPs were degraded from the initial level. This might be due to the strong photostability of CS-NPs, which prevent UV radiation from degrading encapsulated HHC. Many studies have reported that the photostability of various active compounds was significantly enhanced by encapsulation within CS-NPs due to their shielding effect and physical barrier, which can scatter and attenuate the incidence of UV light radiation [14,79].

On the other hand, Suvarna et al. [20] suggested that the rapid adsorption of serum proteins on the surface of nanocarriers results in the formation of a protein corona that may stimulate the immune response or cause activation of the coagulation factors. Thus, the stability of HHC-CS-NPs in BSA, a model protein, was tested to assess the potential utility of nanocarriers in in vivo applications, as shown in Figure 4d. Although the size of HHC-CS-NPs after 24 h of incubation in BSA was 1.6-fold larger (~437 nm) than the initial size (~260 nm), no particle aggregation or dissociation was observed by TEM analysis, as shown in Figure 4e. Furthermore, the EE was reduced by approximately 12% compared to the initial level, indicating that HHC-CS-NPs have good protein stability.

### 3.6. In Vitro Bioaccessibility and Bioavailability

**Figure 5:**
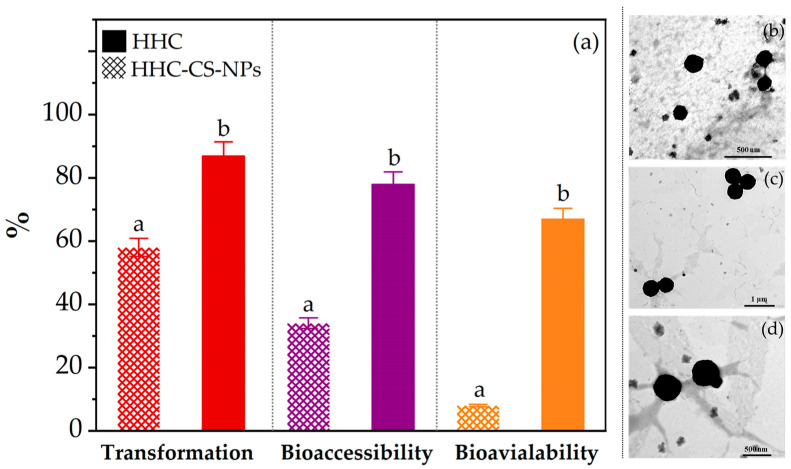
(**a**) Transformation, bioaccessibility, and in vitro bioavailability of HHC in free form (HHC solution) or in HHC-CS-NPs after passing through the simulated GI model (*n* = 3); TEM images of HHC-CS-NPs after passing through simulated GI models: (**b**) oral phase (at 25,000× magnification), (**c**) gastric phase (at 8000× magnification), and (**d**) intestinal phase (at 15,000× magnification). Different letters indicate significant differences at *p* < 0.05.

Each free HHC or HHC-CS-NPs sample was then individually passed through a three-step simulated gastrointestinal tract (GI) model, which included the oral, gastric, and small intestine stages. The in vitro bioavailability was assessed by determining the total amount of HHC remaining, including the fraction in the mixed micelle phase. The results were used to calculate the transformation, bioaccessibility, and bioavailability of HHC. As shown in Figure 5a, the transformation of the HHC-CS-NPs (82.3 ± 1.7%) was significantly higher (*p* < 0.05) than that of the HHC solution (58.3 ± 2.3%), indicating that the CS-NPs could improve HHC stability against pH and digestive enzyme degradation in the simulated GI model. In addition, this finding was correlated with the results of the digestion stability (Figure 5b–d), which indicates the excellent digestion stability of the HHC-CS-NPs. The same trend of in vitro bioaccessibility was observed. The bioaccessibility of the HHC-CS-NPs (78.1 ± 1.3%) was significantly higher (*p* < 0.05) than that of the free HHC (35.4 ± 2.4%), which indicates the higher availability for absorption (bioavailability) of the HHNPs than the free HHC. As expected, the in vitro bioavailability of the HHC-CS-NPs (64.3 ± 1.9%) was six times higher than that of the free HHC (10.7 ± 1.5%), which correlated with the bioaccessibility results. The findings might imply that encapsulating HHC within the HHC-CS-NPs greatly increased its in vitro bioavailability, owing to a large increase in bioaccessibility. In other words, the capability to preserve the structural integrity of the NPs against the degradation by the digestive enzymes present in the simulated GI fluids was evaluated by changes in the structural morphology after passing through each phase of the simulated GI model by TEM analysis, as shown in Figure 5b–d. The analysis results suggested that the HHC-CS-NPs maintained their structural integrity with no disintegration of particles to a small fraction, which implied that the digestive enzymes presented in the simulated GI fluids did not influence the morphology of the HHC-CS-NPs. Overall, it might be suggested that the CS-NPs showed good digestion stability and provided good protection for the active compounds sensitive to harsh GI conditions, which may enhance the bioaccessibility and bioavailability of HHC.

### 3.7. In Vitro Antioxidant Activity

**Figure 6:**
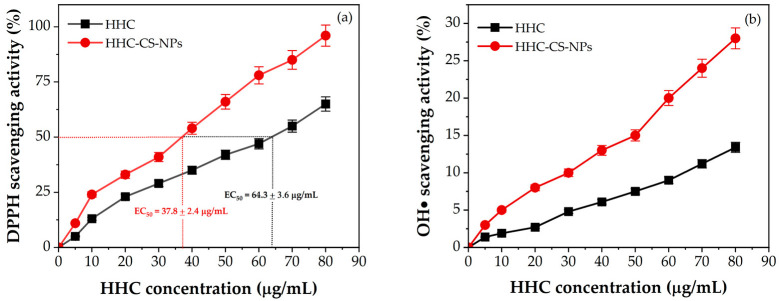
In vitro antioxidation activity of HHC-CS-NPs and HHC solution assessed by (**a**) DPPH radical scavenging assay and (**b**) hydroxyl radical scavenging activity (*n = 3*).

Figure 6a shows the DPPH radical scavenging activity of the different concentrations of HHC in the HHC-CS-NPs or in the HHC solution compared to the empty NPs (CS-NPs). The results showed that the CS-NPs had non-significant (*p* ˃ 0.05) scavenging activity of the CS-NPs (˂3% of activity), implying that the empty CS-NPs had no scavenging activity, whereas the HHC-CS-NPs or free HHC solution had significant scavenging activity (*p* ˂ 0.05) in a concentration-dependent manner. The highest scavenging activity was found in the HHC-CS-NPs at 80 μg/mL (~96%), which was 1.5 times higher than the free HHC solution at the equivalent HHC concentration, indicating that encapsulating HHC in the CS-NPs significantly enhanced its scavenging activity. This may be explained by the fact that the solubility, dispersibility, and stability of HHC were enhanced by encapsulation within the CS-NPs, which resulted in higher antioxidant activity. Furthermore, the half-maximal effective concentration (EC_50_) value of the HHC-CS-NPs (~38 μg/mL) was significantly lower *(p* ˂ 0.05) than that of the free HHC solution (~64 μg/mL), indicating that the encapsulation in CS-NPs enhanced the HHC antioxidant activity. To confirm the antioxidant effect of the HHC-CS-NPs, the scavenging activity of the HHC-CS-NPs toward other free radicals such as hydroxyl radicals (OH•) was needed. The OH• scavenging activity of blank CS-NPs, HHC-CS-NPs, and free HHC solution on OH• radical was found to follow the same trend as the DPPH scavenging activity results, as shown in Figure 6b. The highest OH• scavenging activity (~29%) was found in the HHC-CS-NPs at 80 µg/mL, which was 2.1 times higher than the HHC *(p* ˂ 0.05) at the equivalent HHC concentration, indicating that encapsulation within CS-NPs significantly enhanced the HHC antioxidant activity.

### 3.8. In Vitro Anti-Inflammatory Activity

**Figure 7:**
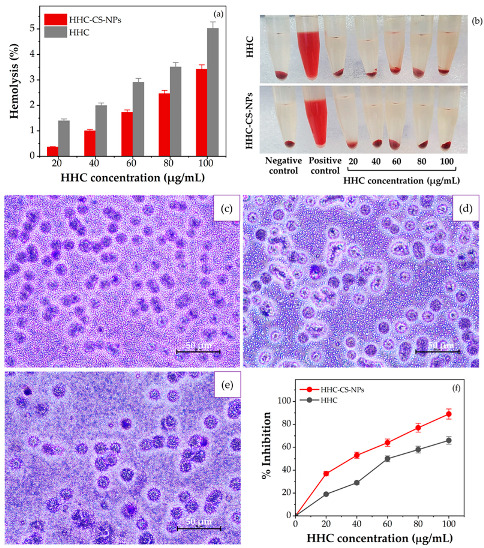
In vitro inflammatory activity assessed by (**a**) hemolysis percentage in Wistar rat erythrocytes following 2 h incubation with HHC or HHC-CS-NP samples with different concentrations of HHC at 37 °C (*n* = 3). (**b**) Image of samples with different concentrations of HHC after centrifugation at 3500× *g* rpm for 5 min: PBS and ultrapure water were negative (−) and positive (+) controls, respectively. The presence of large amounts of hemoglobin in the supernatant is only observed in the positive control; (**c**–**e**) Giemsa staining of Wistar rat erythrocyte smears of (**c**) negative control (PBS), (**d**) HHC, and (**e**) HHC-CS-NPs at the equivalent of 100 μg/mL of HHC concentration. The magnification was ×40. (**f**) Effect of HHC and HHC-CS-NPs on protein denaturation (*n* = *3*).

The RBC membrane is an analogous model membrane for the lysosomal membrane, which plays an important role in releasing inflammatory mediators when inflammation occurs [80]. The anti-inflammatory therapeutic agents that prevent the lysis of inflammatory cell membranes finally decrease the risk and symptoms of inflammation [81]. To assess the in vitro anti-inflammation activity of HHC-CS-NPs or free HHC solution, the hemolysis protection activity assay through the RBC membrane stabilization on hypotonic stress-induced Wistar rat erythrocytes was performed. The hemolysis percentage of the HHC-CS-NPs and free HHC solution was concentration-dependent, as shown in Figure 7a,b, and the highest hemolysis percentage of the HHC-CS-NPs and free HHC solution was about ~2.8 and ~4.5%, respectively, at the equivalent concentration of 100 ug/mL, indicating non-hemolytic and good hemolysis protection activity (˃95%) of all the samples. According to the ISO/TR 7406 guidelines, a hemolysis rate lower than 5% (the critical safe hemolytic ratio for biomaterials) is considered non-hemolytic and required for materials having potential in biomedical applications [82]. Interestingly, the hemolysis protection activity of the HHC-CS-NPs was 1.8 times higher than that of the free HHC solution, suggesting that the hemolysis protection activity of HHC was significantly enhanced by the encapsulation within the CS-NPs compared to the free HHC solution. Similar observations were also reported by Malathy and Priya [83]. To affirm the non-hemolytic activity, the morphology of the RBCs treated with HHC-CS-NPs or free HHC solution was examined under a 40× magnification optical microscope (Figure 7c–e). Interestingly, the morphology, size, distribution, and density of the RBCs treated with the HHC-CS-NPs (Figure 7e) or HHC (Figure 7d) were not different from those in the control group (Figure 7c). In addition, the aggregation of the RBCs treated with the HHC-CS-NPs or free HHC solution was not observed, which may suggest that the RBCs were not affected by the sample groups and may affirm the hemocompatibility and poor toxicity of the HHC-CS-NPs and free HHC solution. Our findings agreed with the studies reported by El-Mekawy and Hudson [84]. The ability of the HHC-CS-NPs or free HHC solution to inhibit BSA denaturation was also investigated to determine the mechanism of anti-inflammatory activity, as shown in Figure 7f. Protein denaturation is well documented in the literature and is the cause of inflammation [85]. According to the results, the percentage of the inhibition of BSA denaturation was concentration-dependent, with the highest inhibition effect observed at the highest concentrations of either the HHC-CS-NPs (87%) or the HHC (~66%), respectively. These results agreed with the studies reported by Malathy and Priya [83]. Our findings may suggest that the inhibition of the protein denaturation effect of HHC was enhanced when it was encapsulated into the CS-NPs.

### 3.9. In Vitro Cytotoxicity

To assess the anticancer activity of the HHC-CS-NPs, free HHC solution, and empty CS-NPs, the MDA-MB-231 cells were separately treated with each sample. The percentage of cell viability was then determined by MTT assay. Generally, toxicity-related NPs have been demonstrated to function in a dose-dependent manner. Thus, using a suitable dose of NPs in a cytotoxicity assay is key to understanding the toxicity effects of the NPs under real-world practical conditions [86].

**Figure 8:**
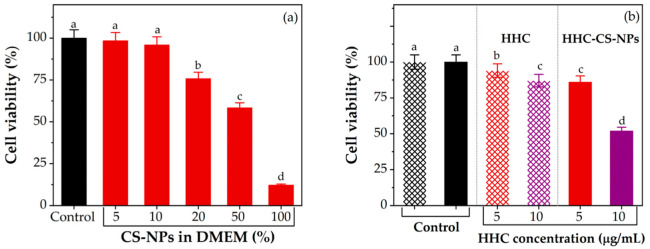
In vitro cytotoxicity expressed as a percentage of cell viability of MDA-MB-231 cells in an MTT assay of (**a**) empty CS-NPs and (**b**) free HHC solution and HHC-CS-NPs. Values are represented as mean ± SD. Bars with the same letters indicate that they are not significantly different from each other (*p* < 0.05).

Herein, the non-toxicity dose of empty NPs (CS-NPs) was primarily determined to choose the suitable dose for the cytotoxicity assay of the HHC-CS-NPs or free HHC solution in the further step. Following the results, as shown in Figure 8a, the viability of the cells treated with CS-NPs was significantly decreased (*p* ˂ 0.05) in a dose-dependent manner. The viability of the cells treated with 5% or 10% CS-NPs was shown to have a non-significant effect (*p* ˃ 0.05) compared to the control (the cells treated with DMEM medium, 100% viability). The cells treated with ≥20% CS-NPs had a significant effect (*p* ˂ 0.05). Hu et al. [87] suggest that the level of oxidative stress and toxicity in zebrafish embryos significantly increases when exposed to a high concentration of CS-NPs. Therefore, 5% and 10% CS-NPs were considered the non-toxicity doses selected for use in the cytotoxicity assay of the HHC-CS-NPs or HHC solution in further studies. In addition, the cells treated with 10% CS-NPs in DMEM and 0.5% DMSO in DMEM served as controls for the HHC-CS-NPs and the HHC solution, respectively. Herein, both sample groups exhibit significantly reduced cell viability (*p* < 0.05) in a concentration-dependent manner. Compared to the HHC solution, the HHC-CS-NPs show significantly decreased cell viability (*p* < 0.05) by 10% and 30% more than the HHC solution at concentrations of 5 μg/mL and 10 μg/mL, respectively. Interestingly, the HHC-CS-NPs at 5 μg/mL showed no significant difference (*p* > 0.05) in the cell viability with HHC at 10 μg/mL, indicating a highly enhanced cytotoxic effect of HHC by encapsulation within CS-NPs. Overall, it may be concluded that the HHC-CS-NPs were more cytotoxic than HHC.

### 3.10. Western Blot Analysis

**Figure 9:**
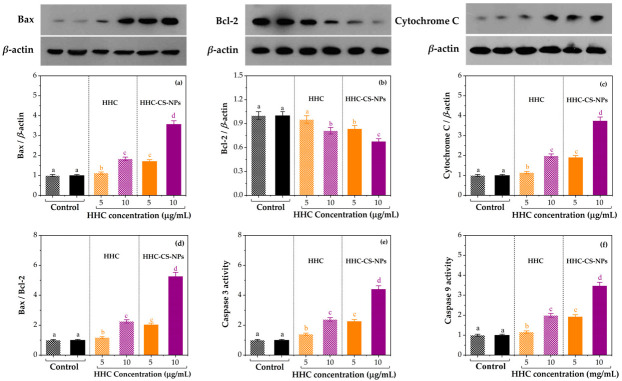
Effects of HHC solution and HHC-CS-NPs on apoptosis-associated protein expression in MDA-MB-231 cells. Expression of (**a**) Bcl-2, (**b**) Bax, (**c**) cytochrome C, (**d**) Bcl-2/Bax ratio, (**e**) caspase-3, and (**f**) caspase-9 protein in MDA-MB-231 cells treated with HHC solution and HHC-CS-NPs at 5 and 10 µg/mL, respectively. *β*-actin, 0.5% DMSO in DMEM and 10% empty CS-NPs in DMEM were used as a positive control, control for HHC solution, and control for HHC-CS-NPs, respectively. Data are represented as mean ± SD. Bars with the same letters indicate that they are not significantly different from each other (*p* < 0.05).

To further fundamentally demonstrate the possible mechanisms of the remarkable anticancer activity of the developed nano-formulation, the four apoptotic (Bax, cytochrome C, caspase 3, and caspase 9) and one anti-apoptotic (Bcl-2) protein expression profiles of the HHC-CS-NPs or HHC solution treated MDA-MB-231 cells were determined using Western blot analysis. β-actin was used as a positive control throughout the study. Herein, the expression of all four apoptotic proteins in the HHC-CS-NP- or HHC-treated cells was significantly increased (*p* ˂ 0.05), while the anti-apoptotic protein was significantly decreased (*p* ˂ 0.05) as compared to the control in a dose-dependent manner (Figure 9). The literature has reported that the mitochondrial-dependent intrinsic pathway of apoptosis is controlled by the Blc-2 protein family [88]. Therefore, the modulation of the cellular expression level of the Blc-2 protein family may be considered as one of the promising approaches to the treatment of many types of cancers [89]. When the Bcl-2 (anti-apoptosis) expression is decreased and the Bax (pro-apoptosis) expression is increased, they cause an imbalance between the Bcl-2 and Bax protein in mitochondria. This leads to the permeability of the mitochondrial membrane changes, resulting in the breakdown and subsequent deterioration of the membrane and the release of cytochrome C from the mitochondria to the cytosol [90]. Then, a cascade reaction occurs in which cytochrome C binds to apoptosis protease activating factor-1 (Apaf-1) in the cytoplasm, activating the initiator caspase-9 and promoting the activation of the effector caspase-3, culminating in cellular apoptosis [91]. The studies reported that HHC could significantly decrease the apoptosis in a rat stork model by the down-regulation in Bax and cleaved caspase-3 expression and the up-regulation in Bcl-2 expression, indicating the anti-apoptosis of HHC in an induced stork rat model [92], which means that HHC may present the apoptosis activities in cancer cells. In this study, significant up-regulated Bax expression and down-regulated Bcl-2 expression (*p* ˂ 0.05) were observed in the HHC-CS-NP- or HHC-treated cells compared to the control, as shown in Figure 9a and Figure 9b, respectively. However, the Bax expression was significantly increased (*p* ˂ 0.05) in the HHC-CS-NP-treated cells and was 2.1 times higher than those of the HHC-treated cells at 10 μg/mL (Figure 9a). In contrast, the Bcl-2 expression was significantly reduced (*p* ˂ 0.05) in the HHC-CS-NP-treated cells, and it was 1.5 and 1.2 times lower than in the control and the HHC-treated cells at 10 μg/mL, respectively (Figure 9b). The literature also reported that the Bax/Bcl-2 ratio was an efficient indicator of the strength of apoptosis [93]. Figure 9c showed that the Bax/Bcl-2 ratio in the HHC-CS-NP-treated cells was 2.3-fold higher than in the HHC-treated cells at 10 µg/mL, indicating that encapsulation in the CS-NPs significantly increased the apoptosis strength of HHC when compared to the free HHC solution. The result was consistent with the previous report that the expression of Bcl-2 in curcumin-loaded CS-NP-treated MCF-7 cells was significantly lower than in the free curcumin-treated cells [94]. Our findings also agreed with the previous study that the Bax/Blc-2 ratio, caspase-3, and caspase-9 expressions in the curcumin/cisplatin-loaded CS-NPs treated in ovarian carcinoma cells were significantly increased when compared with the free curcumin/cisplatin [95]. As mentioned above, the most important steps in the apoptosis process are the release of cytochrome C from the mitochondria into the cytosol. As shown in Figure 9c, the expression of cytochrome C in the cells treated with HHC-CS-NPs was significantly increased (*p* ˂ 0.05). It was 3.8 and 1.5 times higher than in the control and the HHC-treated cells at 10 μg/mL, respectively, suggesting that the encapsulation of HHC in CS-NPs was more effective in causing the release of cytochrome C from the mitochondrial to the cytosol. Chen et al. [96] also demonstrated that the expression of cytochrome C, Bax, Bcl-2, and Bax/Bcl-2 in desmethoxycurcumin/cisplatin-loaded CS-NP-treated non-small cell lung carcinoma cells was significantly higher than that of free desmethoxycurcumin/cisplatin-treated cells. It is well known that the members of the caspase protein family play an important role in apoptosis, especially caspase-3, which is one of the important pro-apoptotic proteins in caspase cascades and is considered a key factor of mitochondrial apoptosis. Moreover, caspase-3 amplifies an executor caspase and the caspase-9 initiation signal by the mitochondrial pathway and cleaves poly (ADP-ribose) polymerase (PARP), thereby amplifying the apoptotic signal [97,98]. As shown in Figure 9e, the expression of caspase-3 in the HHC-CS-NP- or HHC-treated cells was significantly increased compared to the control (*p* ˂ 0.05). The effect of HHC-CS-NPs on the expression of caspase-3 in treated cells was found to have the same trend as the results of the other pro-apoptotic proteins. For example, at 10 µg/mL, the caspase-3 expression was 4.4 and 1.8 times higher in the HHC-CS-NP-treated cells than in the control and the HHC-treated cells, respectively. The effect of HHC-CS-NPs on the expression of caspase-9, another member of the caspase protein family, on the treated cells followed the same trend as the caspase-3 results; at 10 µg/mL, the caspase-9 expression was 3.5 and 1.7 times higher in the HHC-CS-NP-treated cells than in the control and the HHC-treated cells, respectively. Overall, these results suggest that the HHC-CS-NPs exhibited a stronger apoptosis-inducing effect on the MDA-MB-231 cells than the free HHC. However, our present findings on the stability, the in vitro drug release profiles, and the biological activities of HHC (i.e., bioaccessibility, bioavailability, and antioxidant activity) show that it has good stability and controlled and sustained drug release with promising biological activities. In summary, our results suggest that CS-NPs are efficient at enhancing the apoptotic mechanisms, the drug-releasing profile, the stability, and the biological activities of HHC. Therefore, it can be concluded that HHC-CS-NPs may provide mitochondrial-mediated apoptotic activities by improving multifactorial effects.

## 4. Conclusions

HHC-CS-NPs were successfully fabricated and optimized using BBD-RSM to obtain the desired characteristics. The morphology of the optimized HHC-CS-NPs was a spherical shape, and they had a narrow size distribution. The PDI values of the optimized HHC-CS-NPs and CS-NPs (without HHC) were 0.43 ± 0.4 and 0.32 ± 0.2, respectively. The in vitro release profile of HHC from the HHC-CS-NPs under simulated GI and physiological conditions suggested a controlled and sustained release pattern. The HHC-CS-NPs performed well in physicochemical, digestive, and protein stability. The in vitro bioaccessibility, bioavailability, antioxidant activity, anti-inflammatory activity, anti-denaturation of protein activity, and cytotoxicity against MDA-MB-231 breast cancer cells of HHC were significantly enhanced by encapsulation in CS-NPs compared to free HHC. Western blot analysis showed that the HHC-CS-NP-treated cells were significantly up-regulated for the apoptotic proteins and down-regulated for the anti-apoptotic protein compared to the HHC-treated cells, suggesting that mitochondrial-mediated apoptotic activities are present. Overall, it may suggest that the CS-NPs offer promise as potential drug delivery systems for HHC as an alternative therapeutic agent for breast cancer therapy.

## Figures and Tables

**Table 1:** Factors and responses in Box–Behnken design for fabrication of HHC-CS-NPs.

	Level Used		
	Low (−1)	Medium (0)	High (+1)
***Factors***			
*X*_1_ = TPP concentration (mg/mL)	0.6	0.7	0.8
*X*_2_ = Pluronic^®^ F-127 concentration (% *w*/*v*)	0.5	1	1.5
*X*_3_ = HHC concentration (mg/mL)	1	2	3
***Responses***	***Constraints***		
*Y*_1_ = Particle size (nm)	Minimize		
*Y*_2_ = Zeta potential (mV)	*Y*_2_ ≥ 20		
*Y*_3_ = Encapsulation efficiency (%)	Maximize		

**Table 2:** Box–Behnken design matrices with observed response values for development and optimization of HHC-CS-NPs (*n* = 3).

Runs	Factors			Observed Responses		
	*X*_1_	*X*_2_	*X*_3_	*Y*_1_	*Y*_2_	*Y*_3_
1	0.6	0.5	2	102 ± 15	43.8 ± 0.2	67.5 ± 1.3
2	0.8	0.5	2	269 ± 11	24.5 ± 0.5	85.5 ± 2.4
3	0.6	1.5	2	187 ± 17	35.9 ± 0.4	55.8 ± 1.9
4	0.8	1.5	2	387 ± 21	22.2 ± 0.8	83.4 ± 2.2
5	0.6	1.0	1	154 ± 14	41.7 ± 0.3	43.4 ± 1.8
6	0.8	1.0	1	326 ± 18	21.9 ± 0.7	67.3 ± 1.5
7	0.6	1.0	3	198 ± 10	39.7 ± 1.1	74.1 ± 2.3
8	0.8	1.0	3	338 ± 26	20.7 ± 0.4	91.2 ± 0.8
9	0.7	0.5	1	168 ± 12	30.7 ± 0.2	57.5 ± 2.4
10	0.7	1.5	1	275 ± 19	29.6 ± 0.8	49.2 ± 1.1
11	0.7	0.5	3	192 ± 11	30.1 ± 1.2	86.5 ± 2.6
12	0.7	1.5	3	331 ± 22	28.5 ± 0.5	82.8 ± 1.7
13 ^a^	0.7	1.0	2	216 ± 24	31.5 ± 1.6	76.6 ± 1.3
14 ^a^	0.7	1.0	2	230 ± 12	30.4 ± 0.3	75.4 ± 2.8
15 ^a^	0.7	1.0	2	225 ± 17	31.6 ± 0.8	73.4 ± 1.9

^a^ indicates the center point of the design; *X*_1_ = TPP (mg/mL); *X*_2_ = Pluronic^®^ F-127 (% *w*/*v*); *X*_3_ = HHC (mg/mL); *Y*_1_ = Particle size (nm); *Y*_2_ = Zeta potential (mV); and *Y*_3_ = EE (%).

**Table 3:** Optimized composition of HHC-CS-NPs with predicted and experimentally observed values.

Factor	Optimum	Response	Predicted	Observed	% Error
TPP (mg/mL)	0.75	*Y*_1_	250	256 ± 14	2.34
Pluronic^®^ F-127 (% *w*/*v*)	0.50	*Y*_2_	26.8	27.3 ± 0.7	1.83
HHC (mg/mL)	3.00	*Y*_3_	88.7	90.6 ± 1.7	2.09

*Y*_1_ = Particle size (nm); *Y*_2_ = Zeta potential (mV); and *Y*_3_ = EE (%); % Error = (observed value—predicted value)/observed value × 100.

**Table 4:** Kinetic modelling on HHC release from HHCNPs by DDSolver.

Model	Evaluation Criteria						
	Media	*R* ^2^ *_adjusted_*	AIC	MSC	*k_n_*	*n*	*m*
Zero-order (*F* = *k*_0_·*t*)	SBF	0.292	57.668	−0.357	0.667	-	-
	SBF + 1% HSA	0.552	52.946	0.196	0.640	-	-
First-order ($F=100{.\;e}^{{-k}_{1}t}$)	SBF	0.401	56.171	−0.191	0.008	-	-
	SBF + 1% HSA	0.631	51.197	0.391	0.008	-	-
Hixson–Crowell (*F* = 100 [1 − (1 − *k_HC_*·*t*)^3^])	SBF	0.365	56.698	−0.249	0.003	-	-
	SBF + 1% HSA	0.605	51.811	0.322	0.002	-	-
Korsmeyer–Peppas (*F* = *k_KP_*·*t^n^*)	SBF	0.885	42.153	1.367	6.201	0.376	-
	SBF + 1% HSA	0.931	36.875	1.981	4.635	0.446	-
Higuchi (*F* = *k_H_·t*^0.5^)	SBF	0.850	43.664	1.199	4.208	-	-
	SBF + 1% HSA	0.932	35.926	2.087	3.905	-	-
Peppas–Sahlin (*F = k*_1_*·t^m^* + *k*_2_*·t^m^*)	SBF	0.970	30.696	2.639	*k*_1_ = 4.601 *k*_2_ = −0.213	-	0.679
	SBF + 1% HSA	0.969	30.088	2.736	*k*_1_ = 3.522 *k*_2_ = −0.130	-	0.699
Hopfenberg (*F =* 100 [1 − (1 − *k_HB_·t*)*^n^*])	SBF	0.315	58.172	−0.413	0.000	719.798	-
	SBF + 1% HSA	0.578	53.199	0.168	0.002	767.388	-

In all models, *F* is the fraction (%) of drug released in time *t*; *k_n_* is the release constant; and *n and m* are the release exponent of the Korsmeyer–Peppas and Peppas–Sahlin models, respectively.

## Data Availability

All the data are available within the manuscript.

## Supplementary Materials

The following supporting information can be downloaded at: https://www.mdpi.com/article/10.3390/pharmaceutics15020472/s1, Table S1: the summary result of regression analysis for model fitting, Equation (S1): the multiple linear regression for the response particle size (*Y*_1_), Equation (S2): the multiple linear regression for the response zeta potential (*Y*_2_), Equation (S3): the multiple linear regression for the response EE (*Y*_3_). Refs. [99,100] are cited in supplementary materials.
