# Supplementary material for: Potential Oral Anticancer Therapeutic Agents of Hexahydrocurcumin-Encapsulated Chitosan Nanoparticles against MDA-MB-231 Breast Cancer Cells
Source: Pharmaceutics. 2023 Jan 31;15(2):472. doi: 10.3390/pharmaceutics15020472 (PMC9959490; doi:10.3390/pharmaceutics15020472)
Supplement: Supplementary file 1 [file pharmaceutics-15-00472-s001.zip › pharmaceutics-2104577-supplementary.pdf]

Supplementary information

Table S1 Summary result of regression analysis for model fitting.

Response	*p-value*	R^2	$R^2_{adjusted}$	$R^2_{predicted}$	Lack of fit	Remark
Response Y_1 (particle size)						
Linear	< 0.0001	0.9636	0.9537	0.9321	0.1334	Suggested
2FI	0.4999	0.9725	0.9518	0.8954	0.1191	-
Quadratic	0.1048	0.9912	0.9752	0.8741	0.1868	-
Statistically significant factors for Y_1 ($p < 0.05$) are X_1, X_2 and X_3						
Response Y_2 (zeta potential)						
Linear	< 0.0001	0.9563	0.9444	0.9106	0.3358	Suggested
2FI	0.5692	0.9656	0.9398	0.8339	0.2957	-
Quadratic	0.6174	0.9752	0.9306	0.6557	0.2206	-
Statistically significant factors for Y_2 ($p < 0.05$) are X_1, *and* X_2						
Response Y_3 (EE)						
Linear	< 0.0001	0.9435	0.9281	0.8968	0.2042	-
2FI	0.4150	0.9597	0.9294	0.8601	0.1939	-
Quadratic	0.0133	0.9945	0.9846	0.9504	0.6548	Suggested
Statistically significant factors for Y_3 ($p < 0.05$) are X_1, X_2 and X_3						

Notes: X_1 = TPP (mg/mL), X_2 = Pluronic® F-127 (% w/v), X_3 = HHC (mg/mL), Y_1 = particle size (nm), Y_2 = zeta potential (mV), and Y_3 = EE (%).

Equation:

The equation below explained the physical meaning and significance necessary for the second-order polynomial model used in response surface analysis, given as equations S1-S3.

$$Y = \beta_0 + \beta_1 X_1 + \beta_2 X_2 + \beta_3 X_3 + \beta_{12} X_1 X_2 + \beta_{13} X_1 X_3 + \beta_{23} X_2 X_3 + \beta_{11} X_1^2 + \beta_{22} X_2^2 + \beta_{33} X_3^2$$

Where Y is response (dependent variable), β_0 is intercept, β_1 – β_{33} are regression coefficients computed from the observed value of Y from experiments, and X_1, X_2 and X_3 are factors (independent variables). The terms (X_1X_2, X_1X_3, and X_2X_3) and (X_1^2, X_2^2, and X_3^2) represent the interaction and quadratic terms, respectively [1]. The mathematic model equation was used to evaluate the effect of each factor on the responses and determine the optimum setting of these variables to achieve optimum response [2]. "

Equation:

Equation S1 The multiple linear regression for the response particle size (Y_1)

Particle size (Y_1) = + 239.87 + 84.87X_1 + 56.12X_2 + 17.00X_3

Equation S2 The multiple linear regression for the response zeta potential (Y_1)

Zeta potential (Y_2) = + 31.07 - 9.00X_1 – 1.63X_2 - 0.625X_3

Equation S3 The multiple linear regression for the response EE (Y_3)

EE (Y_3) = + 75.00 + 10.62X_1 – 3.25X_2 + 14.63X_3 + 2.51X_1X_2 - 1.75X_1X_3 + 1.50X_2X_3 - 1.12X_1^2 – 0.875X_2^2 - 5.13X_3^2

Where Y is response, X_1 = TPP (mg/mL), X_2 = Pluronic® F-127 (% w/v), X_3 = HHC (mg/mL), Y_1 = particle size (nm), Y_2 = zeta potential (mV), and Y_3 = EE (%).

References

1. Motwani, S.K.; Chopra, S.; Kohli, T.K.; Ahmad, F.J.; Khar, R.K. Chitosan-sodium alginate nanoparticles as submicroscopic reservoirs for ocular delivery: formulation, optimisation and *in vitro* characterisation. *Eur. J. Pharm. Biopharm*, **2008**, *68* (2008), 513–525. doi: 10.1016/j.ejpb.2007.09.009. Epub 2007 Sep 25.
2. Khuri, A.I.; Mukhopadhyay, S. Response surface methodology. W*IREs Comp Stat*, **2010**, *2*, 128-149. doi.org/10.1002/wics.73.
